# Hand Motion Classification Using a Multi-Channel Surface Electromyography Sensor

**DOI:** 10.3390/s120201130

**Published:** 2012-01-30

**Authors:** Xueyan Tang, Yunhui Liu, Congyi Lv, Dong Sun

**Affiliations:** 1 Department of Mechanical and Automation Engineering, The Chinese University of Hong Kong, Hong Kong; E-Mails: yhliu@mae.cuhk.edu.hk (Y.L.); cylv@mae.cuhk.edu.hk (C.L.); 2 Department of Mechanical and Biomedical Engineering, City University of Hong Kong, Hong Kong; E-Mail: medsun@cityu.edu.hk

**Keywords:** surface electromyography, multi-channel, hand motion, concordance correlation, cascaded

## Abstract

The human hand has multiple degrees of freedom (DOF) for achieving high-dexterity motions. Identifying and replicating human hand motions are necessary to perform precise and delicate operations in many applications, such as haptic applications. Surface electromyography (sEMG) sensors are a low-cost method for identifying hand motions, in addition to the conventional methods that use data gloves and vision detection. The identification of multiple hand motions is challenging because the error rate typically increases significantly with the addition of more hand motions. Thus, the current study proposes two new methods for feature extraction to solve the problem above. The first method is the extraction of the energy ratio features in the time-domain, which are robust and invariant to motion forces and speeds for the same gesture. The second method is the extraction of the concordance correlation features that describe the relationship between every two channels of the multi-channel sEMG sensor system. The concordance correlation features of a multi-channel sEMG sensor system were shown to provide a vast amount of useful information for identification. Furthermore, a new cascaded-structure classifier is also proposed, in which 11 types of hand gestures can be identified accurately using the newly defined features. Experimental results show that the success rate for the identification of the 11 gestures is significantly high.

## 1. Introduction

Aside from the conventional sensors and vision methods, the use of biological surface electromyography (sEMG) sensors is a low-cost method for detecting and identifying human motions, such as hand and limb motions. The electrical activity of muscle fibers during a contraction generates the sEMG signals, and then the electrodes attached to the skin record the sEMG signals in a non-invasive manner [1]. The corresponding human motions can be detected and recognized by detecting certain muscle contraction patterns, and the detected motion can be remotely duplicated using artificial limbs or robotic hands [2]. However, the challenge still lies in the detection and recognition phases. Low-cost sEMG sensors have been applied in the rehabilitation field to control prosthetic legs [3] and prosthetic arms [4–10].

In particular, the identification of human hand motions is relatively difficult because the hand has more degrees of freedom (DOF) than the other limbs, and the muscles responsible for finger activation are densely distributed. Current sEMG prosthetic hands in the market are far less dexterous than the human hand, and they are only capable of achieving a limited number of motions, such as hand open and close. Many researchers have concentrated on improving the dexterity of sEMG prosthetic hands [11–14], such that the discrimination of two to six multiple patterns can be achieved. The current study aims to develop an accurate sEMG-based sensing system by describing methods for identifying multiple gestures to reduce the recognition error, which is typically high as the number of predefined gestures increases. Two new methods for feature extraction and a new method for classifier design are proposed to reduce the recognition errors.

The placement of sEMG electrodes is a critical issue for the successful identification of hand motions. Since a user knows in advance the distribution of the corresponding muscles for the hand motions, existing systems are designed such that the sEMG electrodes are pasted on the skin surface right above the corresponding muscles. Thus, identification is highly dependent on proper alignment and failure in doing so results in false identification. Moreover, identification is highly inefficient and inconvenient because users typically have no knowledge about muscle distribution. Recent studies [15,16] have designed and developed multi-channel sensor rings to solve the problem above. The multi-channel sensor ring is a kind of redundant sensor that provides a vast amount of useful information. In the current study, the sEMG sensor is designed as a half wristband covering the posterior side of the forearm, and thus the user can easily wear the sensor ring on the wrist just like a watch.

Feature calculation, which is how useful characteristics from the raw sEMG signals are interpreted, is another critical issue related to successful identification. For traditional placement of sEMG electrodes, the methods for feature extraction include temporal features [17] for the non-complex and low-speed motions and the temporal-spectral features, e.g., short-time Fourier Transform (STFT) and short-time Thompson Transform (STTT), which can provide more transient information for the complex and high-speed movements [12,18,19]. For multi-channel sensor rings, the methods for feature extraction include the extraction of the ratios of temporal- and spectral-features among the different channels [15], and six temporal-features directly used for motion classifier [16]. The advantages of using temporal features include fast computation time and easy implementation. However, temporal methods are force- or speed-sensitive, indicating that their values display large variations when the user moves with different forces or speeds given the same gesture or posture. Given the case that only the type of gesture is of interest, the variations in the temporal features are destructive and they affect the success rate of the identification process. Thus, the first contribution of the current study to solve the problem above is the definition of a new type of measure, namely, the energy ratio feature, which is robust and invariant to different forces or speeds of the same gesture.

As previously mentioned, redundancy is a major feature of multi-channel sEMG sensor rings. The redundant channels of multi-channel sEMG sensor rings generate vast amounts of information, and the manner by which this information is harnessed is a new research issue. Studying the relationship among different channels is one approach for harnessing the information. Recently, researchers have used cross-correlation coefficients to investigate the crosstalk among different channels [20]. Thus, the second contribution of the current study is the in-depth investigation on the relationship of the different channels to define a new concordance correlation feature.

The classifier is another critical issue for the successful identification of hand motions. The classical method uses statistical classification, which is fast and easy to implement for real-time applications. However, the classical method has a low success rate on identifying multiple gestures. Thus, recent studies have investigated artificial neural network classifiers [21,22] and neuron-fuzzy classifiers [23,24]. These advanced classifiers are typically expensive in terms of computation time, and thus they are not feasible for real-time applications. Therefore, the third contribution of the current study is the improvement on the statistical classification method by proposing a new cascaded-structure classifier.

In the following sections, the current study discusses the proposed system and the new methods mentioned above in details. Section 2 introduces the proposed system configuration. Sections 3 and 4 explain the two new extracted features, namely, the energy ratio feature and the concordance correlation feature, respectively. Section 5 discusses the new cascaded-structure classifier. Finally, Section 6 validates experimental results using the newly defined features and classifier in an attempt to identify 11 types of hand gestures.

## 2. System Configuration

**Figure 1.**
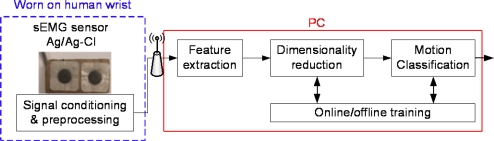
Configuration of the sEMG-based sensing system.

Like other sEMG-based systems, the proposed system consists of four common modules as follows: (1) the sensor ring, (2) signal conditioning and preprocessing, (3) feature extraction, and (4) motion classification, as shown in Figure 1. The sensor ring collects the raw sEMG signals from the skin surface of the human forearm, and the signal conditioning and preprocessing module amplifies and filters the raw sEMG signals with the downsides of miniature amplitude and noise-mixture. The signal conditioning and preprocessing module also converts the conditioned sEMG signals digitally and transfers the digital sEMG signals to a PC via radio frequency (RF) devices. The feature extraction module extracts the representative characteristics from the conditioned sEMG signals, and the classification module identifies the gesture type using the extracted features.

**Figure 2.**
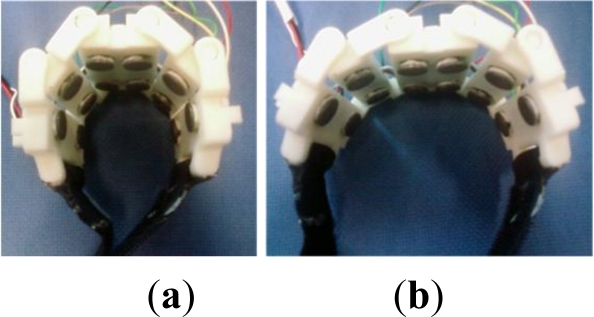
(**a**) Multi-channel sEMG sensor ring for slim forearms. (**b**) Multi-channel sEMG sensor ring for robust forearms.

The sensor ring and the signal conditioning and preprocessing modules were designed to be integrated. The sensor ring was also designed to be compatible with different users, who may have slim or robust forearms, as shown in Figure 2. The sensor ring has six channels of input, and each input channel consists of a pair of sEMG electrodes. The current study explains the need for the six channels of electrodes. Four extensor muscles are known to be responsible for the five-finger movements that are clustered on the posterior side of the forearm. The extensor digitorum is responsible for the movements of the index, middle, ring, and little fingers, and the extensor pollicis longus and brevis are responsible for the thumb. The extensor indicis and extensor digiti minimi are also responsible for the movements of the index finger and the little finger, respectively. Theoretically, four channels are sufficient for recording five-finger movement if the sEMG electrodes are pasted right above the four extensor muscles.

**Figure 3.**
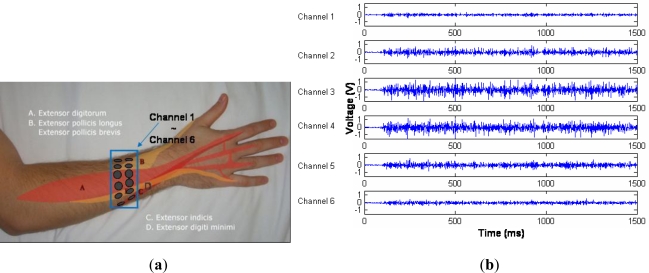
(**a**) Six-channel sEMG electrode arrangement. (**b**) Six-channel conditioned sEMG signals for ball-grasping gesture.

For most users, six channels are enough to cover the circumference of the posterior side. The six channels can record the contraction information of the four extensor muscles and also detect other redundant information. The six-channel of sEMG electrodes are arranged from the index side to the little finger side, as shown in Figure 3(a). Ag/AgCl electrodes by Noraxon with the diameter of 1 cm are used. The distances between every two channels of electrodes are dependent on the forearm sizes of the subjects, because the sensor ring is adjustable for the circumference of the forearm. The distance is relatively large for the robust forearms and small for the slim forearms. Using the six-channel sEMG electrodes, the signals can be recorded at the sampling frequency of 1 kHz. The raw sEMG signals are miniature at the scale from μV and mV, and also noise-mixed. Thus, it is necessary to design the analog amplifiers and filters. To avoid interferences caused by long-wire transmission, the sEMG electrodes were directly fixed on the analog circuits composed of differential amplifiers and filters. The amplifier magnifies the miniature signals to the scale of V. The useful sEMG signals are distributed in the range from 20 to 500 Hz, and thus a two-order high-pass filter for 20 Hz and a two-order low-pass filter for 500 Hz. Moreover, another notch filter is designed for eliminating 50 Hz noise. After conditioned by the analog amplifier and filters, the sEMG signals for ball-grasping gesture are shown in Figure 3(b). Aside from the analog circuits, the signal conditioning and preprocessing module also contains 10-bit A/D converter and RF communication circuits. The amplified and filtered sEMG signals are converted digitally and are transferred to the computer for the following calculation of feature extraction and motion classification. All features are calculated in the time window of 500 ms from the movement starting point.

## 3. New Energy Ratio Feature

The traditional temporal methods for feature extraction include the square integral feature, mean absolute value, and cross-zero rate. These methods have been widely used because they are inexpensive in terms of computation time. However, their values tend to vary even for the same gesture type if the gesture is performed with different forces and speeds, which is considered as one of their major disadvantages. In the cases where only distinguishing the gesture type is of interest, the temporal methods are not too applicable. Recent studies have designed many multi-channel sEMG sensors that have redundant channels to provide more information. The possibility of applying conventional temporal methods of feature extraction to multi-channel sEMG sensors is a new research issue. In a previous research conducted by the current group [25], the new energy ratio feature was defined based on the traditional square integral feature, which is robust to the variations in motion forces and speeds for the same type of hand motion. In the current study, the advantages of the newly defined feature over the traditional square integral feature are discussed and illustrated using an example.

### 3.1. Multi-Channel Energy Ratio Feature

The traditional temporal method based on the square integral feature is given by:

(1) $$E_{i}=\sum_{i=1}^{N}X_{i}^{2}(t),$$

where *i* represents the *i*-th channel of sEMG electrodes, *X_i_*(*t*) is the time-series sEMG signal of the *i*-th channel, and *N* is the data number of the time-series sEMG signal from one channel. Equation (1) essentially describes the absolute energy of one-channel sEMG signals.

The energy ratio feature is defined to get the energy ratio of every two channels. Essentially, the energy ratio feature describes the energy distribution in multiple channels. The ratio of the *i*-th channel to the 1st channel signals is defined as follows:

(2) $${\mathrm{RE}}_{i1}=\frac{E_{i}}{E_{1}},\;\;\;\;\;\;i=2,...,M.$$

All the ratios of the single-channel to the 1st channel signals are defined as follows:

(3) $${\mathrm{RE}}_{1}=\left[\begin{array}{ccc} {\mathrm{RE}}_{21}, & ..., & {\mathrm{RE}}_{M1} \end{array}\right].$$

The ratio of the *i*-th channel to the *j*-th channel signals is represented as follows:

(4) $$\begin{array}{ccc} {\mathrm{RE}}_{\mathrm{ij}}^{*}=\frac{E_{i}}{E_{j}}, & i=2,...,M-1, & j=i+1,...,M. \end{array}$$

The normalization ${\mathrm{RE}}_{\mathrm{ij}}^{*}$ with reference to the 1st channel signal is given by:

(5) $${\mathrm{RE}}_{\mathrm{ij}}=\frac{E_{i}/E_{j}}{E_{j}/E_{1}}=\frac{E_{i}\times E_{1}}{E_{j}^{2}}.$$

The normalization step, which globalizes the ratio of any two channels with the 1st channel as the reference, is important. ${\mathrm{RE}}_{\mathrm{ij}}^{*}$ is still a local ratio of the *i*-th channel to the *j*-th channel signals, which only describes the energy ratio of the *i*-th channel to the *j*-th channel signals. *RE_ij_* is a global parameter that provides the same weight with the 1st channel as the reference. All the ratios of the *i*-th channel to *j*-th channel signals with reference to the 1st channel signal are represented as follows:

(6) $${\mathrm{RE}}_{\mathrm{ij}}=\left[\begin{array}{ccc} {\mathrm{RE}}_{(i+1)i}, & ..., & {\mathrm{RE}}_{Mi} \end{array}\right],\;\;\;\;\;i=2,...,M-1.$$

Combining Equations (3) and (6), the newly defined energy ratio feature can be obtained, with a vector formulated as follows:

(7) $$\mathrm{RE}=\left[\begin{array}{ccccc} {\mathrm{RE}}_{1}, & ..., & {\mathrm{RE}}_{i} & ..., & {\mathrm{RE}}_{M-1} \end{array}\right].$$

where *M* is the channel number, *RE*_1_ is a 1 × (*M* − 1) vector, and *RE_i_* is a 1 × (*M* − 1) vector. Thus, *RE* is a row vector with $\sum_{i=1}^{M-1}\left(M-i\right)$ columns.

In the present experimental case, six channels were used in total, and thus *RE* is a vector of 1 × 15 given by:

(8) $$\mathrm{RE}=\left[\begin{array}{cccccccccccc} {\mathrm{RE}}_{21}, & ..., & {\mathrm{RE}}_{61}, & {\mathrm{RE}}_{32}, & ..., & {\mathrm{RE}}_{62}, & {\mathrm{RE}}_{43}, & ..., & {\mathrm{RE}}_{63}, & {\mathrm{RE}}_{54}, & {\mathrm{RE}}_{64}, & {\mathrm{RE}}_{65} \end{array}\right]$$

### 3.2. Validation of Energy Ratio Feature

**Figure 4.**
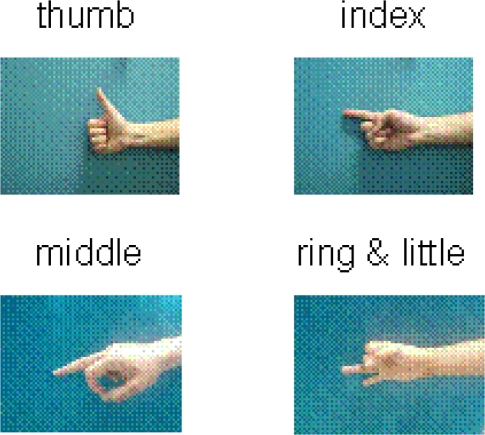
Four gestures: extending thumb, index, middle, and ring and little fingers.

The energy ratio feature was compared with the traditional square integral feature to validate its effectiveness. Using the sensor ring in Figure 2, the sEMG signals were collected from a male subject. The six-channel sEMG signals recorded the activities of the extensor muscles on the posterior side of the forearm. The subject was required to do four gestures, *i.e.*, extending the thumb, index finger, middle finger, and the ring and little fingers simultaneously, as shown in Figure 4.

**Figure 5.**
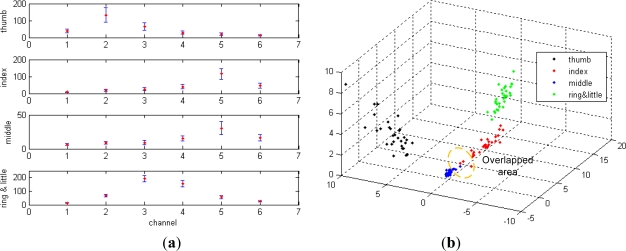
(**a**) Raw square integral features of the four gestures. (**b**) Projected square integral features of the four gestures.

Each type of gesture was repeated 30 times with varying forces and speeds. For each hand motion, the traditional square integral feature using Equation (1) is a 1 × 6 vector. Figure 5(a) shows the averages and variations in the square integral features of the four gestures.

**Figure 6.**
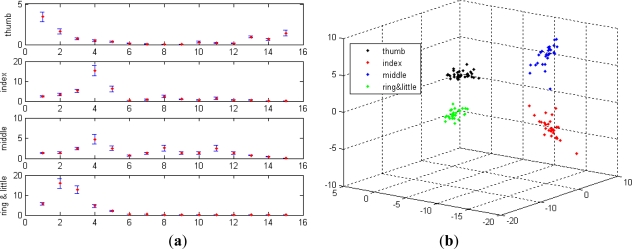
(**a**) Raw energy ratio features of the four gestures. (**b**) Projected energy ratio features of the four gestures.

For each type of gesture, the square integral features fluctuated around the average within the large boundaries formed by the variance when the motion forces varied. Moreover, if the subject exerts an even larger force on the middle finger and a smaller force on the index finger, the square integral features tend to overlap. Overlap leads to the misclassification between the index and middle fingers. Misclassification caused by the overlapped features is apparently seen in the projected space, as shown in Figure 5(b). The projected space is obtained by transforming the original six-dimensional feature space into the three-dimensional space using the Karhunen-Loeve transform (KLT). Figure 6(a) shows the energy ratio features with averages and variances, which were computed using Equation (8). The application of the energy ratio feature avoids misclassification because they are stably distributed within the relatively narrow boundaries even with changes in the motion forces, as shown in Figure 6(b). As can be seen in the figure, the features were distributed separately, and thus no misclassification occurred.

## 4. New Concordance Correlation Feature

The newly booming multi-channel sEMG sensors have redundant channels that provide a vast amount of information. The manner by which the information provided by the redundant channels is utilized is another new research issue. The cross correlation coefficient, also known as the Pearson’s product-moment coefficient, has been used to investigate crosstalk among channels [20]. However, the cross correlation only measures the extent of the linear relationship between two variables. If two variables have a nonlinear relationship, the value of the cross correlation coefficient is zero, and thus the cross correlation coefficient is risky for evaluating the relationship of two variables. Lin [26] defined another solution, which is the concordance correlation coefficient that measures the agreement between two variables. The concordance correlation coefficient has been widely used in data reproducibility studies [26] and image comparison analysis [27]. In previous research conducted by the current group [25], the new concordance coefficient feature was defined and applied in the automatic relocation of sEMG electrodes. The current study attempts to use the concordance correlation coefficient feature for motion identification.

### 4.1. Concordance Correlation Coefficient

The concordance coefficient investigates the agreement between two signals. The concordance correlation coefficient of the *N*-length variables of *x* and *y* is defined as follows:

(9) $$\rho =\frac{2\sigma_{\mathrm{xy}}}{\sigma_{x}^{2}+\sigma_{y}^{2}+{\left(\mu_{x}+\mu_{y}\right)}^{2}}.$$

where *μ_x_* and *μ_y_* are the means of the two variables, respectively. *μ_y_* has the same formula as *μ_x_* that is given by:

(10) $$\mu_{x}=\frac{1}{N}\sum_{i=1}^{N}x_{i}.$$

where *σ_x_* and *σ_y_* are the variances of the two variables, respectively. *σ_y_* has the same formula as *σ_x_* that is given by:

(11) $$\sigma_{x}^{2}=\frac{1}{N}\sum_{i=1}^{N}{\left(x_{i}-\mu_{x}\right)}^{2}.$$

where *σ_xy_* is the covariance of *x* and *y*. *σ_xy_* is given by:

(12) $$\sigma_{\mathrm{xy}}=\frac{1}{N}\sum_{i=1}^{N}\left(x_{i}-\mu_{x}\right)\left(y_{i}-\mu_{y}\right).$$

### 4.2. Multi-Channel Concordance Correlation Feature

The concordance correlation coefficient was used to define the concordance correlation feature of the multi-channel sEMG sensor. For generalized formulation, the sEMG electrodes in the multi-channel sensor ring were assumed to have a total of M pairs. The *M*-channel sEMG signals were represented by an *N* × *M* matrix of $X=\left[\begin{array}{ccccc} X_{1}, & ..., & X_{i}, & ..., & X_{M} \end{array}\right]$. Each column *X_i_* of an *N*-length vector is the time-series sEMG signal of the *i*-th channel. The concordance correlation coefficient of the *i*-th channel and *j*-th channel is defined as follows:

(13) $$\begin{array}{ccc} R_{X_{i}X_{j}}=\rho_{X_{i}X_{j}}, & i=1,...,M-1, & j=i+1,...,M. \end{array}$$

For each hand motion, a $1\times \sum_{i=1}^{M-1}\left(M-i\right)$ vector *R* of the concordance correlation feature can be obtained as follows:

(14) $$R=\left[\begin{array}{ccccc} R_{1}, & ..., & R_{i}, & ..., & R_{M-1} \end{array}\right],$$

where:

$$\begin{array}{c} R_{1}=\left[\begin{array}{cccc} R_{X_{1}X_{2}}, & R_{X_{1}X_{3}}, & ..., & R_{X_{1}X_{M}} \end{array}\right],\\ R_{i}=\left[\begin{array}{cccc} R_{X_{i}X_{i+1}}, & R_{X_{i}X_{i+2}}, & ..., & R_{X_{i}X_{M}} \end{array}\right],\\ R_{M-1}=\left[R_{X_{M-1}X_{M}}\right]. \end{array}$$

For the multi-channel sEMG sensor, the defined concordance correlation feature essentially describes the homogeneity of every two channels in terms of amplitude and variation. In the current case, the sEMG electrodes have six channels, and the sEMG signals of each hand motion are represented by an *N* × 6 matrix of $X=\left[\begin{array}{cccccc} X_{1}, & X_{2}, & X_{3}, & X_{4}, & X_{5}, & X_{6} \end{array}\right]$. For each hand motion, a 1 × 15 vector *R* of the concordance correlation feature can be obtained as follows:

(15) $$R=\left[\begin{array}{ccccc} R_{1}, & R_{2}, & R_{3}, & R_{4}, & R_{5} \end{array}\right],$$

where:

$$\begin{array}{c} R_{1}=\left[\begin{array}{ccccc} R_{X_{1}X_{2}}, & R_{X_{1}X_{3}}, & R_{X_{1}X_{4}}, & R_{X_{1}X_{5}}, & R_{X_{1}X_{6}} \end{array}\right],\\ R_{2}=\left[\begin{array}{cccc} R_{X_{2}X_{3}}, & R_{X_{2}X_{4}}, & R_{X_{2}X_{5}}, & R_{X_{2}X_{6}} \end{array}\right],\\ R_{3}=\left[\begin{array}{ccc} R_{X_{3}X_{4}}, & R_{X_{3}X_{5}}, & R_{X_{3}X_{6}} \end{array}\right],\\ R_{4}=\left[\begin{array}{cc} R_{X_{4}X_{5}}, & R_{X_{4}X_{6}} \end{array}\right],\\ R_{5}=\left[R_{X_{5}X_{6}}\right]. \end{array}$$

### 4.3. Validation of Concordance Correlation Feature

**Figure 7.**
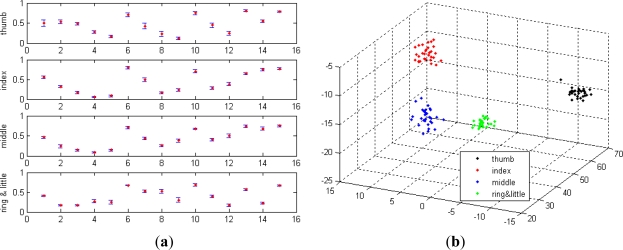
(**a**) Raw concordance correlation features of the four gestures. (**b**) Projected concordance correlation features of the four gestures.

The same experimental sEMG signals for validating the energy ratio feature were used to validate the effectiveness of the concordance correlation feature. Figure 7 shows the concordance correlation features of the four gestures (shown in Figure 4) that were calculated using Equation (15). The concordance correlation features of each type of gesture were shown to be uniform and distributed within the narrow boundaries. Moreover, the concordance correlation features of the different gesture types were different from one another, indicating that the concordance correlation feature contains the discriminatory information for the different gesture types and is applicable for gesture discrimination.

## 5. Cascaded-Structure Classifier

The traditional classification methods are statistical classifiers, such as the linear discriminant analysis (LDA), K-nearest classifier, Bayes classifier and so on. Statistical classifiers have the advantage of fast computation time and they are easy to implement for real-time applications. However, statistical classifiers become less efficient for identification when more gesture types are introduced because the features are projected into another space, and an increase in the number of gesture types will typically produce more overlapping areas for the projecting features. Statistical classification methods create a cluster that contains the features of the same type of gesture or generate a hyperplane to separate the different gestures. Therefore, misclassification occurs when there are overlapping areas between different gestures.

Avoiding the overlapping areas between different gestures in the projected space is the solution to make statistical classification methods applicable for identifying more gestures. The proposed classifier divides the classification procedure into several levels. In each level, the different features and the different projected spaces, which contain most discriminatory information for the gestures included in the level, are located.

The development of the cascaded-classifier can be concluded in several steps. In the first step, all types of hand motions are regarded as individuals. The newly defined energy ratio feature can be used in this level, which represents the energy distribution in the six-channel sEMG electrodes. In this step, the hand motions are projected into the reduced-dimensional space and are classified as several separable groups. Each group may include only one type of hand motion or several types of motions. Group separation is based on the rule that there are the similar energy ratio features within the same group, and the different energy ratio features among the different groups.

In the second step, each separable subgroup is classified independently. If the subgroup includes several types of hand motions, these included types of hand motions are regarded as individuals. The features need to be recalculated using other methods because the features used in the upper-classifier have less discriminatory information for the subgroup. For example, the energy ratio feature is used in the upper-level classifier, and it means that the gestures in the subgroup have similar energy distribution information. In this level, the concordance correlation feature, which represents the different agreements between the channels, can be used to recalculate the features. The features are then transformed into a new space because the old space in the upper-level classifier has the best views for the separable subgroups but not for the gestures in the subgroup. In this new space, the types of hand motions in the subgroup are distributed as separately as possible, and the second-level classifier is designed. If the subgroups still include several types of hand motions, the second step is repeated in the sub-subgroups until every type of hand motion can be identified separately. The concordance correlation features are still used in this level, and the new projection space is found by the rule of best discriminatory view in the subgroups.

## 6. Results and Discussion

**Figure 8.**
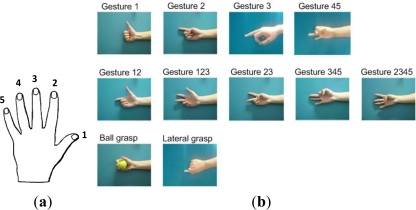
(**a**) Finger label. (**b**) Eleven predefined gestures.

The experimental results are presented in this section to validate the effectiveness of the newly defined features and the proposed cascaded-structure classifier for identifying more types of gestures. Eleven types of gestures were defined and six male subjects were selected for the experiment. Each finger was labeled using numbers 1 to 5, as shown in Figure 8(a), and the 11 gestures were named using the same rule, as shown in Figure 8(b). The extensions of the individual fingers are defined as the basic movements, *i.e.*, gestures 1, 2, 3, and 45, as shown in Figure 8(b). Gesture 45 is defined as the basic movement when the ring and little finger always move together. Gestures 12, 123, 23, 345, and 2345 can be regarded as the combined movements of the basic gestures. Moreover, two types of grasping movements were defined, *i.e.*, ball grasp and lateral grasp. Each subject was required to repeat each type of gesture 30 times, and 25 samples were used to design the classifier, and the other 5 samples were used to test the designed classifier. Each subject wore the sensor ring shown in Figure 2 on the forearm, and the six-channel sEMG electrodes recorded the sEMG signals of the extensor muscles distributed on the posterior side of the forearm.

**Figure 9.**
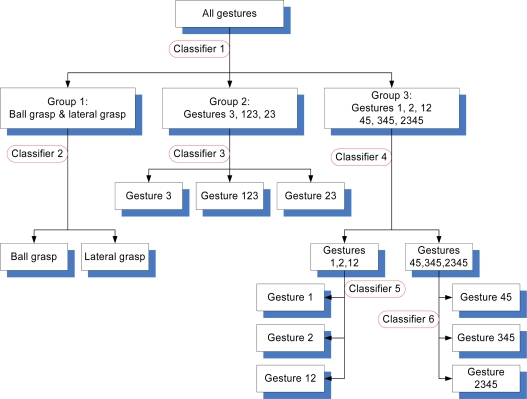
Configuration of the cascaded-classifier of the first subject.

The sEMG signals are influenced by many factors, such as muscle distribution, forearm size, and finger coordination, among others. Thus, different people will generate different sEMG signals. Although the sEMG signals were different for different subjects, the development of the classifiers followed the same steps instructed in the previous section, where different subjects will have different cascaded-classifier structures. The current study discusses the development of the cascaded-classifier for one subject in details. Figure 9 and Table 1 show the configuration of the designed cascaded-classifier for the first subject.

**Figure 10.**
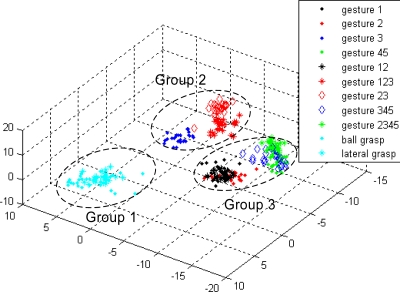
Classifier 1 in the top-level of the first subject.

Initially, the 11 gestures were regarded as individual types. In the top-level classification, the energy ratio features of the 11 gestures were calculated. For each hand motion, the energy ratio feature was a 1 × 15 vector. Since each type of gesture was repeated 25 times for the classifier design, the energy ratio feature of each type of gesture was a matrix of 25 × 15. The energy ratio feature of the 11 types of gesture is a matrix of 275 × 15. The 275 × 15 feature matrix should be dimensionally reduced initially by projecting it into another space before designing the classifier. The necessity for such projection is supported by two reasons. The first reason is that each energy ratio feature is 15-dimensional, and thus the computation would be expensive if the 15-dimensional feature is directly used for designing the classifier. The second reason is that the 15-dimensional feature spaces of all types of gestures have no optimal views for classification, that is, the features are not separated as possible from the other types. Thus, instead of the conventional principal component analysis (PCA), the method of KLT [28] was used to dimensionally reduce the feature matrix and find the best space for type separation and to transform the features from 275 × 15 to 275 × 3. The transform matrix was determined using the rule of large separation among the basic gestures 1, 2, 3, and 45. Seven other types of gestures were transformed into the three-dimensional space above. All 11 gestures can be classified into three groups using LDA (Figure 10), which is the detail for designing Classifier 1.

**Figure 11.**
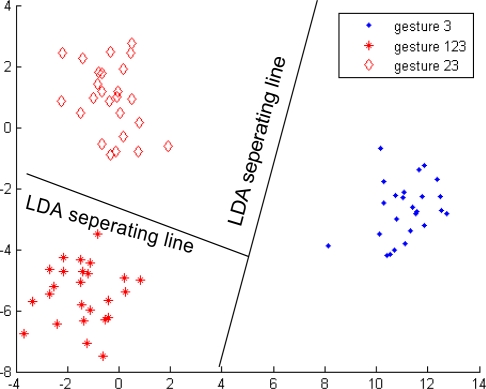
Classifier 3 in the second-level of the first subject.

Up to this point, we still cannot uniquely identify any individual hand movement. Therefore, second-level classifiers, namely, Classifier 2, Classifier 3, and Classifier 4, were continuously being developed. The current study discusses Classifier 3 as an example, and Classifiers 1 and 3 were developed in the same way. Gestures 3, 123, and 23 were included in Group 2. Two problems need to be addressed in the development of Classifier 3. The first problem is defining the feature describing the difference among gestures 3, 123, and 23. Gestures 3, 123, and 23 have similar energy ratio features, making the energy ratio feature not suitable for distinguishing among these three gestures. The second problem is finding the projected space in which gestures 3, 123, and 23 are located using the rule of largest separation among them. The concordance correlation feature was used to solve the first problem. Gestures 3, 123, and 23 were regarded as individuals, and KLT was used to find the projected space in which gestures 3, 123, and 23 will have the largest separation and solve the second problem. Gestures 3, 123, and 23 can be correctly and individually grouped using Classifier 3, as shown in Figure 11.

**Figure 12.**
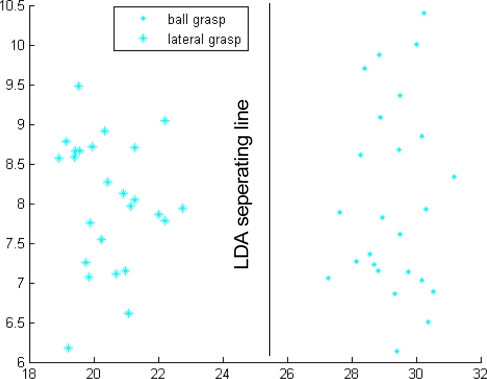
Classifier 2 in the second-level of the first subject.

**Figure 13.**
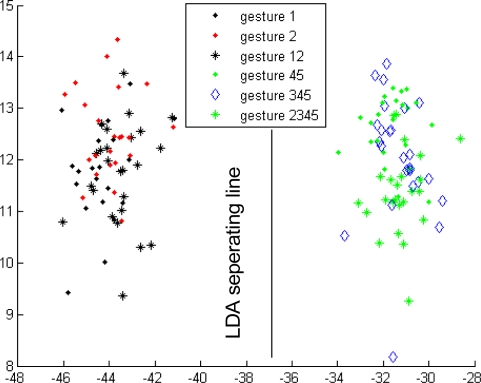
Classifier 4 in the second-level of the first subject.

**Figure 14.**
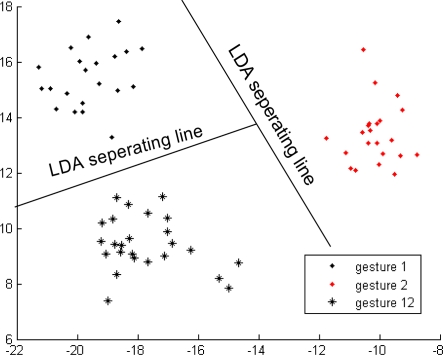
Classifier 5 in the third-level of the first subject.

**Figure 15.**
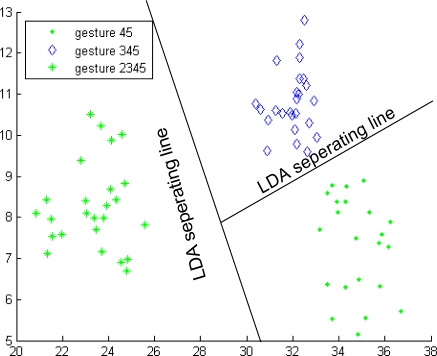
Classifier 6 in the third-level of the first subject.

Similarly, the gestures of ball grasp and lateral grasp can be correctly classified using Classifier 2, as shown in Figure 12. Gestures 1, 2, 12, 45, 345, and 2345 were regarded as two separate types when Classifier 4 was developed. The first type includes gestures 1, 2, and 12, and the other type includes gestures 45, 345, and 2345. The same procedures as those in Classifier 3 were repeated and the two types can be correctly grouped, as shown in Figure 13. Moreover, the third-level Classifiers 5 and 6 were continuously developed, and Figures 14 and 15 show the classification results. Classifier 5 can identify gestures 1, 2, and 12, and Classifier 6 can distinguish gestures 45, 345, and 2345. At this point, the cascaded-classifier was achieved.

The subject was required to repeat each type of gesture five times. A total of fifty-five hand motions for the 11 types of gestures were used as the test data. Table 2 lists the success rate of the 11-gesture classification. Among the 55 hand motions, one gesture 1 was misclassified as gesture 2. For all of the 55 test gestures, the error rate was only 1 out of 55 gestures, and thus the success rate was about 98%. The identification procedure of the gesture is implemented in Visual C++ program, and results show that the computation cost is low. The identification time is approximately 172 ms for one new gesture.

As comparison, the conventional LDA classifier is also developed for the first subject. Similarly, totally 11 gestures are defined, and 25 trials for each type of gesture are used as the training data. All types of gestures are regarded as individual and projected in one 3-D space, and LDA classifier is designed in this projected space. Similarly 55 hand motions are used for the test set, and the success rate was only 46%. The low success rate is because there are many overlapped areas among the different types of gestures in the projected space. The method of our cascaded classifier avoids the case of the overlapped features in the projected space.

The development of the classifiers for the other five subjects followed the same procedure as the first subject. Tables 3 to 7 list the classification results of the other five subjects, respectively. The classification results of the six subjects show that the two new features and the new cascaded-classifier are effective for identifying more types of gestures.

## 7. Conclusions

The identification of hand motions becomes more difficult as the number of hand motion types increases. The identification success rate decreases significantly when more types of hand motions are added. The current study solves this problem by defining new features and designing a new cascaded-structure classifier. In the different levels of the cascaded-classifier, the different features, including the newly defined features, were projected onto the different spaces of the classifier design. The cascaded-classifier avoided the overlapping areas in the projected space that usually occur using conventional classification methods. The experimental results show that the proposed cascaded-classifier and the new features are effective for identifying more types of gestures, with the success rate of the 11-gesture identification being greater than 89%.

## Figures and Tables

**Table 1.** Cascaded-classifier of the first subject.

**Classifier**	**Type**	**Projected Space**	**Feature**

Classifier 1	LDA	KLT using gestures 1, 2, 3, and 45 as bases	Energy ratio feature
Classifier 2	LDA	KLT using ball grasp and lateral grasp as bases	Concordance correlation feature
Classifier 3	LDA	KLT using gestures 3, 123, and 23 as bases	Concordance correlation feature
Classifier 4	LDA	KLT using the combination of gestures 1, 2, and 12 and the combination of gestures 45, 345, and 2345 as bases	Concordance correlation feature
Classifier 5	LDA	KLT using gestures 1, 2, and 12 as bases	Concordance correlation feature
Classifier 6	LDA	KLT using gestures 45, 345, and 2345 as bases	Concordance correlation feature

**Table 2.** Success rate of the 11-gesture classification of the first subject.

**Gesture**	1	2	3	45	12	123	23	345	2345	Ball	Lateral
**Success No. / Test No.**	4/5	5/5/	5/5	5/5	5/5	5/5	5/5	5/5	5/5	5/5	5/5
**Total success rate**	98.2%										

**Table 3.** Success rate of the 11-gesture classification of the second subject.

**Gesture**	1	2	3	45	12	123	23	345	2345	Ball	Lateral
**Success No. / test No.**	5/5	5/5/	5/5	5/5	5/5	5/5	3/5	4/5	5/5	5/5	5/5
**Total success rate**	94.5%										

**Table 4.** Success rate of the 11-gesture classification of the third subject.

**Gesture**	1	2	3	45	12	123	23	345	2345	Ball	Lateral
**Success No. / test No.**	4/5	5/5/	5/5	5/5	5/5	5/5	5/5	4/5	5/5	5/5	5/5
**Total success rate**	96.4%										

**Table 5.** Success rate of the 11-gesture classification of the fourth subject.

**Gesture**	1	2	3	45	12	123	23	345	2345	Ball	Lateral
**Success No. / test No.**	5/5	5/5/	5/5	5/5	5/5	5/5	5/5	4/5	5/5	5/5	5/5
**Total success rate**	98.2%										

**Table 6.** Success rate of the 11-gesture classification of the fifth subject.

**Gesture**	1	2	3	45	12	123	23	345	2345	Ball	Lateral
**Success No. / test No.**	5/5	4/5/	5/5	5/5	5/5	5/5	4/5	5/5	3/5	5/5	5/5
**Total success rate**	89.1%										

**Table 7.** Success rate of the 11-gesture classification of the sixth subject.

**Gesture**	1	2	3	45	12	123	23	345	2345	Ball	Lateral
**Success No. / test No.**	5/5	4/5/	5/5	5/5	5/5	5/5	3/5	4/5	5/5	5/5	5/5
**Total success rate**	92.7%										

## References

[1] Merletti R., Parker P. (2004). Electromyography: Physiology, Engineering and Noninvasive Applications.

[2] Cipriani C., Antfolk C., Controzzi M., Lundborg G., Rosen B., Carrozza M.C., Sebelius F. (2001). Online Myoelectric Control of a Dexterous Hand Prosthesis by Transradial Amputees. IEEE Trans. Neural Syst. Reh. En.

[3] Jin D.W., Zhang R.H., Zhang J.C., Wang R.C., Gruver W.A. An Intelligent Above-Knee Prosthsis with EMG-Based Terrain Identification.

[4] Ito K., Tsuji T., Kato A., Ito M. (1992). EMG Pattern Classification for a Prosthetic Forearm with Three Degrees of Freedom.

[5] Doringer J.A., Hogan N. (1995). Performance of Above Elbow Body-Powered Prostheses in Visually Guided Unconstrained Motion Tasks. IEEE Trans. Biomed. Eng.

[6] Saridis G.N., Gootee T.P. (1982). EMG Pattern Analysis and Classification for a Prosthetic Arm. IEEE Trans. Biomed. Eng.

[7] Castellini C., Gruppioni E., Dayalli A., Sandini G. (2009). Fine Detection of Grasp Force and Posture by Amputees via Surface Electromyography. Neurorobotics.

[8] Castellini C., Smagt P. (2008). Surface EMG in Advanced Hand Prosthetics. Biol. Cybern.

[9] Peerdeman B., Boere D., Witteveen H., in 't Veld R.H., Hermens H.J., Stramigioli S., Rietman H., Veltink P.H., Misra S. (2011). Myoelectric Forearm Prostheses: State of the Art from a User-Centered Perspective. J. Rehabil. Res. Dev.

[10] Nielsen J.L.G., Holmgaard S., Jiang N., Englehart K.B., Farina D., Parker P.A. (2011). Simultaneous and Proportional Force Estimation for Multifunction Myoelectric Prostheses Using Mirrored Bilateral Training. IEEE Trans. Biomed. Eng.

[11] Hudgins B., Parker P. (1993). A New Strategy for Multifunction Myoelectric Control. IEEE Trans. Biomed. Eng.

[12] Farry K.A., Walker I.D., Barabiuk R.G. (1996). Myoelectric Teleoperation of a Complex Robotic Hand. IEEE Trans. Robotics Automat.

[13] Fukuda O., Tsuji T., Kaneko M. An EMG Controlled Robotics Manipulator using Neural Networks.

[14] Kuribayashi K., Okimura K., Taniguchi T. A Discrimination System using Neural Network for EMG-Controller Prostheses—Integral Type of EMG Signal Processing.

[15] Saponas T.S., Tan D., Morris D., Balakrishnan R. Demonstrating the Feasibility of Using Forearm Electromyography for Muscle-Computer Interfaces.

[16] Du Y.-C., Lin C.-H., Shyu L.-Y., Chen T. (2010). Portable Hand Motion Classifier for Multi-Channel Surface Electromyography Recognition using Grey Relational Analysis. Expert Syst. Appl.

[17] Zecca M., Micera S., Carrozza M.C., Dario P. (2002). Control of Multifunctional Prosthetic Hands by Processing the Electromyographic Signal. Crit. Rev. Biomed. Eng.

[18] Hannaford B., Lehman S. (1986). Short Time Fourier Analysis of the Electromyogram: Fast Movements and Constant Contraction. IEEE Trans. Biomed. Eng.

[19] Du S.J., Vuskovic M. Temporal *vs.* Spectral Approach to Feature Extraction from Prehensile EMG Signals.

[20] Mogk P.M., Keir J. (2003). Crosstalk in Surface Electromyography of the Proximal Forearm during Gripping Tasks. J. Electromyograph. Kinesiol.

[21] Haykin S. (1999). Neural Networks: A Comprehensive Foundation.

[22] Micheli-Tzanakou E. (2000). Supervised and Unsupervised Pattern Recognition: Feature Extraction and Computational Intelligence.

[23] Micera S., Sabatini A., Dario P., Rossi B. (1999). A Hybrid Approach for EMG Pattern Analysis for Classification of Arm Movements using Statistical and Fuzzy Techniques. Med. Eng. Phys.

[24] Abe S., Lan M. (1995). A. Method for Fuzzy Rule Extraction Directly from Numerical Data and Its Application to Pattern Classification. IEEE Trans. Fuzzy Syst.

[25] Tang X.Y., Liu Y.H, Lu C.Y., Poon W.L. (2011). Classification of Hand Motion using Surface EMG Signals. Biologically Inspired Robotics.

[26] Lin I.-K. (1989). A Concordance Correlation Coefficient to Evaluate Reproducibility. Biometrics.

[27] Lange N., Strother S.C., Anderson J.R., Nielsen F.A., Holmes A.P., Kolenda T., Savoy R., Hansen L.K. (1999). Plurality and Resemblance in fMRI Data Analysis. Neuroimage.

[28] Webb A.R. (2002). Statistical Pattern Recognition.

